# Post-transplantation Cyclophosphamide: From HLA-Haploidentical to Matched-Related and Matched-Unrelated Donor Blood and Marrow Transplantation

**DOI:** 10.3389/fimmu.2020.00636

**Published:** 2020-04-09

**Authors:** Louis Williams, Frank Cirrone, Kelli Cole, Maher Abdul-Hay, Leo Luznik, Ahmad Samer Al-Homsi

**Affiliations:** ^1^Division of Hematology and Medical Oncology, NYU Langone Health, New York, NY, United States; ^2^Blood and Marrow Transplantation Program, NYU Langone Health, New York, NY, United States; ^3^Division of Oncology, Johns Hopkins University, Baltimore, MD, United States

**Keywords:** GvHD prevention, post-transplant cyclophosphamide, matched-related donor, matched unrelated donor, bortezomib, calcineurin inhibitor-free

## Abstract

Following allogeneic blood and marrow transplantation (BMT), graft-versus-host disease (GvHD) continues to represent a significant cause of treatment failure, despite the routine use of conventional, mainly calcineurin inhibitor-based prophylaxis. Recently, post-transplant cyclophosphamide (PTCy) has emerged as a safe and efficacious alternative. First, omitting the need for *ex vivo* T-cell depletion in the setting of haploidentical transplantation, growing evidence supports PTCy role in GvHD prevention in matched-related and matched-unrelated transplants. Through improved understanding of GvHD pathophysiology and advancements in drug development, PTCy emerges as a unique opportunity to design calcineurin inhibitor-free strategies by integrating agents that target different stages of GvHD development.

## Introduction

Despite continued improvement in the outcomes of allogeneic blood and marrow transplant (BMT) over the last decade, the prospects of acute and chronic graft-versus-host disease (aGvHD and cGvHD) continue to drive treatment-related mortality (TRM) and limit the utility and wide applicability of this valuable treatment modality (1). Conventional combinations of calcineurin (CN) or mammalian target of rapamycin (mTOR) inhibitors, coupled with either methotrexate (MTX) or mycophenolate mofetil (MMF), achieve rates of aGvHD and cGvHD of approximately 40–75% and 40–70% following matched-related donor (MRD) and matched-unrelated donor (MUD) transplants (2). In addition to their partial efficacy, these regimens target T-cells broadly and indiscriminately, therefore delaying immune reconstitution and hampering graft-versus-leukemia (GvL) effect. Furthermore, both CN inhibitors (CNI) and mTOR inhibitors (mTORI) have a narrow therapeutic index with multiple drug interactions rendering prescriber experience and patients’ compliance essential for their safety and efficacy (3). Lastly, as these agents are administered for 6 to 9 months, they typically prevent early post-transplant introduction of interventions and small molecules aimed to decrease the risk of disease relapse.

Post-transplant cyclophosphamide (PTCy), initially developed to overcome human leukocyte antigen (HLA) barriers in the setting of haploidentical transplantation (4), has proved promising following MRD and MUD transplants (5–7). Furthermore, PTCy may represent an ideal platform for the development of CN and mTORI-free GvHD prevention strategies.

## Mechanisms of PTCy In GVHD Prevention

Cyclophosphamide is an alkylating agent that acts through its metabolites, phosphoramide and acrolein, to induce DNA strand breakage that ultimately leads to replication stress in rapidly dividing cells (8). The efficacy of cyclophosphamide appears to be cell-cycle dependent and is highest in the G1 and S phases (9). In studies evaluating the specific effects of cyclophosphamide on cytotoxic T-cell lines, Strauss et al. observed that increased apoptosis, mediated by increased Fas expression, may differentiate cyclophosphamide from other immunosuppressive agents (4, 10, 11).

The biological underpinnings of PTCy-induced immune tolerance have yet to be fully elucidated. However, there is evidence to support that PTCy eliminates alloreactive T-cell clones of both donor and host origin in the early post-transplantation period with relative preservation of regulatory T-cells (12, 13). This is supported by early evidence from murine skin allograft experiments where allografted mice were treated with cyclophosphamide shortly after engraftment. In these test animals, donor-derived alloreactive T-cell populations were eliminated via extra-thymic mechanism, presumably related to cyclophosphamide administration (14, 15). Simultaneously, regulatory T-cells were selectively spared, possibly due to their expression of a specific aldehyde dehydrogenase that confers resistance to cyclophosphamide (16, 17). Life-long immune tolerance was subsequently maintained by central, intra-thymic clonal deletion of the anti-host T-cells derived from donor hematopoietic stem cells.

The previously demonstrated pivotal role of regulatory T-cells in PTCy-induced immune tolerance was recently corroborated by a series of experiments performed by Waschmuth et al. Mice treated with PTCy at 25 mg/kg on day +3 and +4 following haploidentical transplantation showed significantly less severe GvHD than mice treated with 5 or 100 mg/kg a day. In these experiments, immune tolerance developed despite the persistence of alloreactive T-cells following optimally dosed PTCy and in the absence of thymus. Rather than eliminating alloreactive T-cells, PTCy induced functional impairment of these cells, supported by robust suppressive mechanisms that included rapid and preferential recovery of regulatory T-cells (18, 19). Further validation of these mechanisms will improve our understanding of PTCy-induced immune tolerance and identify the optimal dosing of cyclophosphamide.

## PTCy in Haploidentical Transplant

An early study established that PTCy can overcome HLA barriers and omit the need for *ex vivo* T-cell depletion following a non-myeloablative preparative regimen and haploidentical bone marrow transplant (20, 21). In this trial, the investigators compared one dose of PTCy administered on day +3 and two doses of PTCy on day +3 and +4, demonstrating a decreased incidence of cGvHD in the group receiving two doses (25% versus 5%, *p* = 0.05%) with no differences in aGvHD, event-free survival (EFS) or overall survival (OS). The incidence of grades II-IV and III-IV aGvHD for the entire cohort was 34 and 6%, respectively (21). This data was confirmed in a larger study conducted by Kasamon et al. and Munchel et al. In a cohort of 210 patients, the incidence of grades II-IV acute and cGvHD were 27 and 13%, respectively (22, 23). The rates of disease relapse, EFS, and OS were 55, 35, and 27%, respectively.

Following the initial studies, which were focused on bone marrow as the graft source, the safety and efficacy of PTCy-based GvHD prevention were validated following both myeloablative and non-myeloablative conditioning regimens by several investigators. In a study by Solomon et al. using busulfan-based myeloablative conditioning, the incidence of grade II-IV and III-IV aGvHD and cGvHD were 30, 10, and 35% (24). Similar results were reported by Bhamidipati et al. following a non-myeloablative preparative regimen (25). More recently, Wang et al. showed that the addition of low dose PTCy (14.5 mg/kg on day +3 and +4), to the so-called Beijing protocol, reduced the incidence of grade II-IV aGvHD and improved GvHD- and relapse-free survival (GRFS) (26).

Based on these studies and others, haploidentical transplantation has become, with the advent of PTCy, one of the most commonly used alternative donor strategies. Multiple retrospective comparisons have demonstrated similar overall survival of haploidentical transplantation to that of HLA-matched donor and cord blood transplants (27).

## PTCy as Monotherapy in MRD and MUD Transplant

Since establishing its role in the haploidentical setting, several investigators examined the applicability of PTCy to GvHD prevention in MRD and MUD transplants. Luznik et al. reported the incidence of acute and cGvHD following myeloablative conditioning in 117 recipients of MRD (*n* = 78) and MUD (*n* = 39) bone marrow grafts. In this study, GvHD prophylaxis consisted of a single agent PTCy. Grades II-IV and III-IV acute GvHD rates were 43 and 10% for the entire cohort. The long-term incidence of cGvHD was particularly low at 10%. EFS sand OS were 55 and 39% (5). Interestingly, 43% of patients did not require any other form of immunosuppressive therapy (28). These favorable results were corroborated by a similar multi-institutional trial by Kankary et al. with a low incidence of cGvHD at 14% (6).

Unfortunately, when Alousi et al. examined PTCy as the only GvHD prophylaxis following reduced-intensity preparative regimen and peripheral blood grafts, the results were strikingly different (29). In this study, 38 patients received bone marrow and 11 patients received peripheral blood grafts. Twenty-two patients received rabbit anti-thymocyte globulin (rATG) before the study was amended to omit rATG. The rates of grade II-IV and grade III-IV aGVHD were of 58% and 22%, whereas the rate of cGVHD was 18%. When the authors compared the results to a matched, historical cohort of patients receiving TAC and MTX for GvHD prophylaxis, significantly higher rates of all grades of aGvHD [46% vs. 19%, hazard ratio (HR) = 2.8, *p* = 0.02] as well as inferior TRM (HR = 3.3, *p* = 0.035) and OS (HR = 1.9, *p* = 0.02) were observed in the PTCy cohort. There were no differences in cGvHD between the prospectively treated patients and the historical control (29). Similarly, unsatisfactory results were reported in a smaller phase II study by Holtick et al. (30). The authors examined the safety and efficacy of PTCy as monotherapy for GvHD prevention following reduced-intensity conditioning and MRD and MUD peripheral blood transplants. The rate of TRM was unacceptably high at 36%, principally attributable to an increased rate of severe intestinal aGVHD. A study by Bradstock et al. was terminated early when four out of the first five patients developed life-threatening aGvHD, two of whom died (31).

Cumulatively, the current evidence suggests that, while single-agent PTCy may represent a viable prophylactic option in patients receiving myeloablative conditioning and bone marrow graft, it is inadequate in the setting of reduced-intensity conditioning and peripheral blood transplantation.

## PTCy and CNI or mTOR Inhibitors in MRD and MUD Transplant

Given the shortcomings of PTCy as monotherapy for GvHD prophylaxis following MRD and MUD peripheral blood transplants, several groups reverted to combining PTCy with a CNI or mTORI with or without MMF, aiming to reduce the relatively high incidence of cGvHD characteristic of CNI and mTOR inhibitors-based combinations. To this end, Mierlcarek et al. combined PTCy with cyclosporine A (CSA) in patients receiving myeloablative conditioning. The rates of grades II-IV and III-IV aGvHD were favorable at 77 and 0% and again with a low incidence of cGvHD at 16%. The rates of TRM and disease relapse were 14 and 17% (32). Moiseev et al. compared the outcomes of patients receiving a combination PTCy, TAC and MMF to the outcomes of consecutive historical control patients receiving TAC, MMF and rATG following myeloablative conditioning and MUD or 1–2 HLA loci mismatched-unrelated donor peripheral blood grafts. The rates of grades II-IV and III-IV aGvHD were 19% vs. 45% (*p* = 0.003) and 4% vs. 27%, (*p* < 0.001), respectively. The incidence of cGvHD was 16% vs. 65% (*p* < 0.001). EFS and OS were also improved in the PTCy group (HR = 0.49, 95% CI 0.31–0.78, *p* = 0.006, and 0.43, 95% CI 0.26–0.7, *p* = 0.007) (33). Carnevale-Schianca et al. employed the same GvHD prevention regimen in 35 patients receiving RIC and MRD, MUD, or 1 HLA locus mismatched-unrelated donor peripheral blood transplants. The patients achieved grades II-IV aGvHD and cGvHD rates of 17 and 7% with no grade IV aGvHD. The 2-year TRM rate was 3% with EFS and OS rates of 54% and 77% (34).

Two studies examined the combination of PTCy and sirolimus. Solomon et al. conducted a phase II study that included 26 patients treated with RIC following MUD and peripheral blood transplants. Sirolimus was stopped without taper between day +90 and 100. The rates of grade III-IV acute and cGvHD were higher than the aforementioned rates with PTCy and CNI combinations at 16 and 31% (35). Greco et al. elaborated on the use of PTCy in combination with sirolimus in 28 patients receiving a myeloablative preparative regimen and MRD or MUD peripheral blood allografts. MMF was added to the regimen in patients receiving MUD transplants. The incidence of grades II-IV acute and cGvHD seemed better at 23 and 13% (36).

The most substantial evidence favoring a PTCy-based GvHD prevention strategy in the setting of MRD or MUD donor transplants stems from a recent Blood and Marrow Transplant Clinical Trial Net randomized phase II trial (37). In this trial patients received RIC and were randomized to one of three GvHD prevention regimens: TAC, MTX, and bortezomib, TAC, MTX and maraviroc or PTCy, TAC, and MMF. Patients with MRD, MUD, or 1 HLA locus mismatched-unrelated donors were included. Each of the trial’s three groups was then compared to a contemporaneous prospective control group receiving TAC and MTX from non-participating institutions. Comorbidities were more frequent in the control group. The distribution of the conditioning regimens was also different. Among the three groups, only the group treated with PTCy-based prophylaxis had better outcomes in comparison to the control cohort. The rates of grades II-IV and III-IV aGvHD for the PTCy group were 27% (90% CI 20%–35%) and 2% (90% CI 0–5%). The corresponding rates in the control group were 30% (90% CI 25%–36%) and 13% (90% CI 9–16%). The 1-year incidence of cGvHD was 28% (90% CI 20%–36%) and 28% (90% CI 33%–43%), respectively. The 1-year GRFS rates were also superior in the PTCy group (HR = 0.72, 95% CI 0.54–0.94, *p* = 0.044). However, there was no difference in TRM, DFS, and OS. These results were corroborated by a prospective randomized trial comparing CSA and MMF to PTCy and CSA following MRD and MUD peripheral blood transplants. The group receiving PTCy-based GvHD prophylaxis had lower rates of acute and cGvHD and improved GRFS (38).

In summary, pending the results of an ongoing phase III randomized trial (CTN03959241), comparing PTCy in combination with TAC and MMF to TAC and MTX, PTCy in combination with a CNI for GvHD prophylaxis may potentially emerge as a new standard of care for the prevention of GvHD in the setting of MRD and MUD transplantation.

## PTCy as a Platform for CN and mTOR Inhibitor-Free GvHD Prevention in MRD and MUD Transplant

Given the previously mentioned pitfalls of CN and mTOR inhibitor-containing GvHD prevention regimens, our work over the last several years focused on exploiting PTCy in order develop CN and mTOR inhibitor-free GvHD preventive combinations.

Proteasome inhibitors have multiple immune modulatory effects that span different stages of GvHD development including dendritic and T-cell differentiation, proliferation and function. Proteasome inhibitors also foster the expansion of regulatory T-cells (39, 40). Despite the fact that the results of a recent phase II trial examining the combination of bortezomib with CN and mTORI compared to a standard TAC and MTX combination were disappointing (41), we hypothesized based on pre-clinical data that proteasome inhibitors remain appealing agents when paired with PTCy. In a murine model, the combination of PTCy and ixazomib resulted in superior survival of animals subjected to lethal GvHD in comparison to either drug alone. Furthermore, PTCy prevented the surge in interleukin-1β (IL-1β) and donor T-cell expansion characteristic of delayed administration of proteasome inhibitors following transplantation. The combination induced profound post-transplant cytokine suppression including IL-6, IL-1β, and tumor necrosis factor-α (42). In a clinical trial, bortezomib was added to PTCy in MRD and MUD transplantation following RIC and peripheral blood grafts. Patients receiving MUD transplantation also received r-ATG. Two doses of bortezomib were given 6 h after graft infusion and 72 h thereafter. All GvHD prophylaxis was completed on day +4. The rates of aGvHD grades II to IV and III to IV were 35.9% (95% CI 18.6–53.6%) and 11.7% (95% CI 2.8%–27.5%). The rate of cGvHD was 27% (95% CI 11.4%–45.3%). The 2-year GRFS was 37.7% (95% CI 20.1%–55.3%) (43). When compared to a registry control group the 1-year GRFS was 39% (95% CI 24%–54%) in the study group and 32% (95% CI 27%–38%) in the control group (HR = 0.81, 90% CI 0.52–1.27, *p* = 0.44) (unpublished data). These promising results are being confirmed in a larger trial.

Table 1 provides a summary of selected registered studies that use PTCy-based GvHD prevention in MRD and MUD transplantation.

**TABLE 1:** Selected registered studies of PTCy-based GvHD prevention in MRD and MUD transplantation.

**Study identification**	**Study type**	**Intervention**	**Responsible party**
NCT04202835	Phase III, Randomized	rATG versus rATG and PTCy	Sarah Kleiboer
NCT04232085	Phase II	PTCy, tacrolimus, and MMF in patients with primary bone marrow failure or immunodeficiency syndromes	Orly Klein
NCT03357159	Phase II	PTCy and rATG	Arnon Nagler
NCT02629120	Phase II	PTCy and sirolimus in patients with chronic granulomatous disease	Elizabeth Kang
NCT02861417	Phase II	PTCy, tacrolimus and MMF	Uday Popat
NCT03192397	Phase II	PTCy, sirolimus and MMF	Christine Ho
NCT03818334	Phase III, Randomized	rATG, CNI, and MMF versus PTCy, CNI and MMF	Andreza Feisoa Ribeiro
NCT03945591	Phase II	PTCy, bortezomib and rATG	A Samer Al-Homsi
NCT03602898	Phase II	CNI and MTX versus CNI, MTX, and rATG or PTCy and CNI	Masumi Ueda
NCT03959241	Phase III, Randomized	Tacrolimus and MTX versus PTCy, tacrolimus and MMF (GvHD prevention and stool microbiome)	Mary Horowitz
NCT03555851	Phase I	PTCy (pharmacogenetics predictors of efficacy)	Chojecki, Aleksander
NCT03246906	Phase II	Cyclosporine, sirolimus, and MMF versus PTCy, cyclosporine, and sirolimus	Masumi Ueda
NCT03263767	Phase II	PTCy	Patrice Cehvallier
NCT04160390	Phase I	PTCy (biomarkers predictors of efficacy)	Jeannine McCune
NCT03680092	Phase II	Tacrolimus and MTX versus PTCy and abatacept	Divya Koura
NCT02556931	Phase II	PTCy, tacrolimus (short course) and MMF	Amy E. Dezern
NCT02876679	Phase II	Cyclosporine, MMF and rATG versus PTCY, cyclosporine, and MMF	Mohamad Mothy
NCT02833805	Phase II	PTCy, tacrolimus, and MMF in patients with severe aplastic anemia	Amy E. Dezern

*rATG, rabbit anti-thymocyte globulin; MMF, mycophenolate mofetil; MTX, methotrexate.*

## Future Directions

**FIGURE 1:**
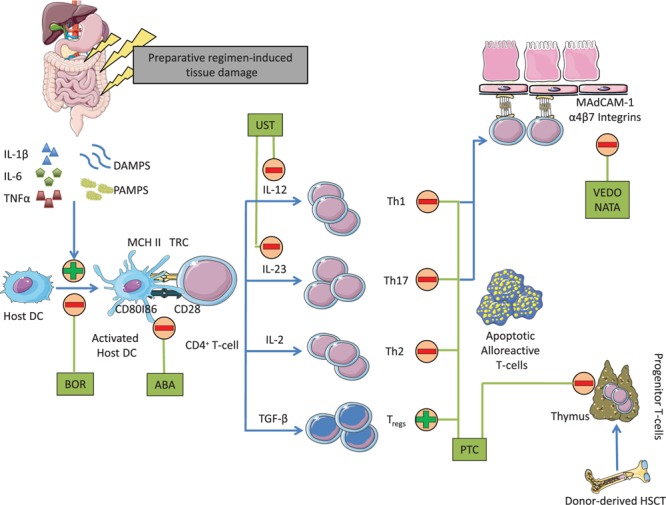
Mechanism of action of agents that may conceptually be added to PTCy to develop CN and mTORI-free combinations for GvHD prevention. DAMPS, damage-associated molecular patterns; PAMPS, pathogen-associated molecular patterns; BOR, bortezomib; ABA, abatacept; MHC II, major histocompatibility complex class II; UST, ustekinumab; TGF-β, transforming growth factor-β; HSC, hematopoietic stem cells; VEDO, vedolizumab; NATA, natalizumab.

Improving upon our understanding of GvHD pathophysiology and our advancement in drug development offer additional opportunities to rationally design CN and mTORI-free PTCy-based GvHD prevention combinations following MRD and MUD transplantation. Toward this end, T-cell co-stimulation blockade, integrin antagonists and IL-23 inhibitors seem attractive as these agents target different phases of GvHD development (Figure 1). Abatacept, a soluble fusion compound of cytotoxic T-cell associated antigen-4 (CTLA-4) and immunoglobulin G1 (IgG1) that binds to CD80 and prevents dendritic cells from delivering a second stimulation signal for T-cell activation, reduced the incidence of aGvHD when added to a standard combination of CNI and MTX in patients receiving matched or one HLA locus mismatched-unrelated donor transplantation (44). Anti-integrin therapy, on the other hand, prevents T-cell trafficking into the guts. Vedolizumab, which acts on a gut-trophic α4β7 integrin and natalizumab, which acts on α4-integrin are being examined in GvHD prevention and treatment (45, 46). Lastly, IL-23 subunit p40 antagonist, ustekinumab, polarizes T-cell differentiation thus preventing the development of T-helper 1 (Th1) and T-helper 17 (Th17) and favoring the expansion of regulatory T-cells is also being studied in GvHD prevention (47). Other IL-23 p19 subunit inhibitors including guselkumab, tildrakizumab and risankizumab may also be of interest. Carefully designed clinical trials are warranted to examine the potential role of these agents in the prevention of GvHD.

## Conclusion

Since establishing its role in HLA-haploidentical transplantation, PTCy has emerged as an effective platform in GvHD prevention strategies in MRD and MUD transplantation. Pending ongoing randomized study, PTCy in combination with TAC and MMF may represent a new standard of care based on its ease of administration and efficacy. Furthermore, PTCy offers a unique opportunity for the development of CN and mTORI-free GvHD preventive combinations, allowing an early introduction of immune manipulations and small molecules aimed to prevent disease relapse following allogeneic BMT.

## Author Contributions

All authors contributed to the writing of the manuscript.

## Conflict of Interest

AA-H received research support from Millennium Pharmaceuticals. The remaining authors declare that the research was conducted in the absence of any commercial or financial relationships that could be construed as a potential conflict of interest.

## References

[1] Singh AK McGuirk JP. Allogeneic stem cell transplantation: a historical and scientific overview. *Cancer Res.* (2016) 76:6445–51. 10.1158/0008-5472.CAN-16-1311 27784742

[2] Ram R Gafter-Gvili PV Yeshurun M Paul M Raanani P Shpilberg O. Prophylaxis regimens for GvHD: systemic review and meta-analysis. *Bone Marr Transplant.* (2009) 43:643–53. 10.1038/bmt.2008.373 18997826

[3] Przepiorka D Nash RA Wingard JR Zhu J Maher RM Fitzsimmons WE Relationship of tacrolimus whole blood levels to efficacy and safety outcomes after unrelated donor marrow transplantation. *Biol Blood Marrow Transplant.* (1999) 2:94–7. 10371361 10.1053/bbmt.1999.v5.pm10371361

[4] Luznik L O’Donnell PV Fuchs EJ. Post-transplantation cyclophosphamide for tolerance induction in HLA-haploidentical BMT. *Semin Oncol.* (2012) 39:683–93. 10.1053/j.seminoncol.2012.09.005 23206845 PMC3808078

[5] Luznik L Bolaños-Meade J Zahurak M Chen AR Smith BD Brodsky R High-dose cyclophosphamide as single-agent, short course prophylaxis of graft-versus-host disease. *Blood.* (2010) 115:3224–30. 10.1182/blood-2009-11-251595 20124511 PMC2858487

[6] Kanakry CG O’Donnell PV Furlong T de Lima MJ Wei W Medeot M Multi-Institutional study of post-transplantation cyclophosphamide as a single-agent graft-versus-host disease prophylaxis after allogeneic bone marrow transplantation using myeloablative busulfan and fludarabine conditioning. *J Clin Oncol.* (2014) 32:3497–505. 10.1200/JCO.2013.54.0625 25267759 PMC4209101

[7] El Fakih R Hashmi S Ciurea SO Luznik L Gale RP AlJurf M. Post-transplant cyclophposphamide use in matched HLA donors: a review of literature and future application. *Bone Marr Transplant.* (2020) 55:40–7. 10.1038/s41409-019-0547-8 31089284

[8] Whirl-Carrillo M McDonagh EM Hebert JM Gong L Sangkuhl K Thom CF Pharmacogenomics knowledge for personalized medicine. *Clin Pharmacol Therap.* (2012) 92:414–7. 10.1038/clpt.2012.96 22992668 PMC3660037

[9] Emadi A Jones RJ Bodsky RA. Cyclophospahmide and cancer: golden anniversay. *Nat Rev Clin Oncol.* (2009) 6:638–47. 10.1038/nrclinonc.2009.146 19786984

[10] Strauss G Osen W Debatin KM. Induction of apoptosis and modulation of activation and effector function in T cells by immunosuppressive drugs. *Clin Exp Immunol.* (2002) 128:255–66. 10.1046/j.1365-2249.2002.01777.x 11985515 PMC1906394

[11] Al-Homsi AS Roy T Cole K Feng Y Duffner U. Post-transplant high-dose cyclophosphamide for the prevention of graft-versus-host disease. *Biology of Blood and Marrow Transplant.* (2014) 21:604–11. 10.1016/j.bbmt.2014.08.014 25240817

[12] Mussetti A Greco R Peccatori J Corradini P. Post-transplant cyclophosphamide, a promising anti-graft versus host disease prophylaxis: where do we stand? *Exp Rev Hemtol.* (2017) 10:479–92. 10.1080/17474086.2017.1318054 28395546

[13] Cieri N Peccatori J Oliveira G Greco R Marktel S Lunghi F Tracking T cell dynamics in the first month after haploidentical HSCT with post-transplant cyclophosphamide reveals a predominant contribution of memory stem T cells to the early phase of immune reconstitution. *Blood.* (2013) 122:4615 10.1182/blood.v122.21.4615.4615

[14] Eto M Mayumi H Tolmita Y Yoshikai Y Nishimura Y Maeda T Specific destruction of host-reactive mature T cells of donor origin prevents graft-versus-host disease in cyclophosphamide-induced tolerant mice. *J Immunol.* (1991) 146:1402–9. 1671578

[15] Huyan XH Lin YP Gao T Chen RY Fan YM. Immunosuppressive effect of cyclophosphamide on white blood cells and lymphocyte subpopulations from peripheral blood of Balb/c mice. *Int Immunopharmacol.* (2011) 11:1293–7. 10.1016/j.intimp.2011.04.011 21530682

[16] Kanarky C Ganguly S Zahurak M Bolaños-Meade J Thoburn C Perkins B Aldehyde dehydrogenase expression drives human regulatory t cell resistance to posttransplant cyclophosphamide. *Sci Transl Med.* (2013) 5:211 10.1126/scitranslmed.3006960 PMC4155575 24225944

[17] Ganguly S Ross DB Panoskaltsis-Mortari A Kanakry CG Blazar BR Levy RB Donor-derived CD4+ Foxp3+ regulatory cells are necessary for post-transplantation cylophosphamide-mediated protection against GVHD in mice. *Blood.* (2014) 124:2131–41. 10.1182/blood-2013-10-525873 25139358 PMC4186542

[18] Wachsmuth LP Patterson M Eckhaus MA Venzon DJ Gress RE Kanakry CG. Posttransplantation cyclophosphamide prevents graft-versus-host disease by induced alloreactive T cell dysfunction and suppression. *J Clin Invest.* (2019) 129:2357–73. 10.1172/JCI124218 30913039 PMC6546453

[19] Radjocic V Luznik L. Mechanism of action of posttransplantation cyclophosphamide: more than meets the eye. *J Clin Inves.* (2019) 129:2189–91. 10.1172/JCI128710 31063990 PMC6546449

[20] O’Donnell PV Luznik L Jones RJ Vogelsang GB Leffell MS Phelps M Nonmyeloablative bone marrow tranaplantation from partially HLA-matched related donors using posttransplantation cyclophosphamide. *Biol Blood Marr Transplant.* (2002) 8:377–86. 10.1182/bloodadvances.2016002766 12171484

[21] Luznik L O’Donnell PV Symons HJ Chen AR Leffell MS Zahurak M HLA-haploidentical bone marrow transplantation for hematologic malignancies using nonmyeloablative conditioning and high-dose posttransplantation cyclophosphamide. *Biol Blood Marr Transplant.* (2008) 14:641–50. 10.1016/j.bbmt.2008.03.005 PMC2633246 18489989

[22] Kasamon YL Luznik L Leffell MS Kowalski J Tsai HL Bolaños-Meade J Nonmyeloablative HLA-haploidentical bone marrow transplantation with high-dose posttransplantation cyclophosphamide: effect of HLA disparity on outcome. *Biol Bood Marrow Transplant.* (2010) 16:482–9. 10.1016/j.bbmt.2009.11.011 19925877 PMC2998606

[23] Munchel A Kesserwan C Symons HJ Luznik L Kasamon YL Jones RJ Nonmyeloablative HLA-haploidentical bone marrow transplantation with high dose post-transplantation cyclophosphamide. *Pediatr Rep.* (2011) 3(Suppl. 2):43–7. 10.4081/pr.2011.s2.e15 PMC3206539 22053277

[24] Solomon SR Sizemore CA Sanacore M Zhang X Brown S Holland HK Haploidentical tranplantation using T-cell replete peripheral blod stem cells and myelaobaltive conditioning in patients with high risk hematologic malignancies who lack conventional donors is well tolerated and produces excellent relapse-free survival: results of prospective phase II trial. *Biol Blood Marr Transplant.* (2012) 18:1859–66. 10.1016/j.bbmt.2012.06.019 22863841

[25] Bhamidipati PK Dipersio JF Stokerl-Goldstein K Rashidi A Gao F Uy GL Haploidentical transplantation using G-CSF-mobilizaed T-cell replete PBSCs and post-transplant CY after non-myelablative conditioning regimen is safe and is assocaited with favorable outcomes. *Bone Marr Transplant.* (2014) 48:1124–26. 10.1038/bmt.2014.108 24842528

[26] Wang Y Wu DP Liu QF Xu LP Liu KY Zhang XH Low-dose post-transplant cyclophophamide and anti-thymocyte globulin as an effective strategy for GVHD prevention in haploidentical patients. *J Hematol Oncol.* (2019) 12:88. 10.1186/s13045-019-0781-y 31481121 PMC6724335

[27] McCurdy SR Luznik L. How we perform haploidentical stem cell transplantation with posttransplant cyclophosphamide. *Blood.* (2019) 134:1802–10. 10.1182/blood.2019001323 31751485 PMC6872960

[28] Kankary C Tsai HL Bolanos-Meade J Douglas Smith B Gojo I Kankary JA Single-agent GVHD prophylaxis with posttransplantion cylophosphamide after myeloablative, HLA-matched BMT for AML, ALL, and MDS. *Blood.* (2014) 124:3817–27. 10.1182/blood-2014-07-587477 25316679 PMC4263989

[29] Alousi A Brammer JE Saliba RM Andersson B Popat U Hosing C *P*hase II trial of GVHD prophylaxis with post-transplanatation cyclophosphamide following reduced-intensity busulfan/fludarabine (Bu/Flu) conditioning for hematologic malignancies. *Biol Blood Marr Transplant.* (2015) 21:906–12. 10.1016/j.bbmt.2015.01.026 PMC4825327 25667989

[30] Holtick U Chemnitz J Shimabukuro-Vornhagen A Theurich S Chakupurakal G Krause A OCTET-CY: a phase II study to investiagte the efficacy of a post-transplant cyclophosphamide as sole graft-versus host prophylaxis after allogeneic peripheral blood stem cell transplantation. *Euro J Hematol.* (2016) 96:27–35. 10.1111/ejh.12541 25703164 PMC4844370

[31] Bradstock KF Bilmon I Kwan J Micklethwaite K Blyth E Deren S Single-agent high-dose cyclophosphamide for graft-versus-host disease prophylaxis in human leukocyte antigen-matched reduced-intensity peripheral blood stem cell transplantation. *Biol Blood Marr Transplant.* (2015) 21:934–53. 10.1016/j.bbmt.2015.01.020 25636379

[32] Mielcarek M Furlong T O’Donnell PV Storer BE McCune JS Storb R Posttransplantation cyclophosphamide for prevention of graft-versus-host disease after HLA-matched mobilized blood cell transplantation. *Blood.* (2016) 127:1502–8. 10.1182/blood-2015-10-672071 26764356 PMC4797026

[33] Moiseev IS Pirogova OV Alyanski AL Babenko EV Gindina TL Darskaya EI Graft-versus-host disease prophylaxis in unrelated peripheral stem cell transplantation with post-transplantation cyclophosphamide, tacrolimus, and mycophenolate mofetil. *Biol Blood Marr Transplant.* (2016) 22:1037–42. 10.1016/j.bbmt.2016.03.004 26970381

[34] Carnevale-Schianca F Caravelli D Gallo S Coha V D’Ambrosio L Vassallo E Post-Transplant cyclophosphamide and tacrolimus-mycophenolate mofetil combination prevents graft-versus-host disease in allogeneic peripheral blood hematopoietic cell transplantation from HLA-matched donors. *Biol Blood Marr Transplant.* (2017) 23:459–66. 10.1016/j.bbmt.2016.12.636 28039079

[35] Solomon SR Sanacore M Zhang X Brown S Holland K Morris LE Calcineurin inhibitor-free graft-versus-host disease prophylaxis with post-transplantation cyclophosphamide and grief-course sirolimus following reduced-intensity peripheral blood stem cell transplantation. *Biol Blood Marr Transplant.* (2014) 20:1828–34. 10.1016/j.bbmt.2014.07.020 25064745

[36] Greco R Lorentino F Morelli M Giglio F Mannina D Assanelli A Posttransplantation cyclophosphamide and sirolimus for prevention of GvHD after HLA-matched PBSC transplantation. *Blood.* (2016) 128:1528–31. 10.1182/blood-2016-06-723205 27495140

[37] Bolanos-Meade J Reshef R Fraser R Fei M Abhyankar S Al-Kadhimi Z Three prophylaxis regimens (tacrolimus, mycophenolate mofetil, and cyclophosphamide; tacrolimus, methotrexate, and bortezomib; or tacrolimus, methotrexate, and maraviroc) versus tacrolimus and methotrexate for prevention of graft-versus-host disease with haemopoietic cell transplantation with reduced-intensity conditioning: a randomised phase 2 trial with a non-randomised contemporaneous control group (BMT CTN 1203). *Lancet Haematol.* (2019) 6:e132–43. 10.1016/S2352-3026(18)30221-7 30824040 PMC6503965

[38] De Jong CN Meijer E Bakunina K Nur E Kooji MVM de Groot M Post-transplantation cyclophosphamide after allogeneic hematopoietic stem cell transplantation: results of the prospective randomized HOVO-96 trial in recipients of matched related and unrelated donors. *Blood.* (2019) 134(suppl. 1):1 10.1182/blood-2019-124659

[39] Mohty M Brissot E Savani BN Gaugler B. Effects of bortezomib on the immune system; a focus on immune regulation. *Biol Blood Marr Transplant.* (2013) 19:1416–20. 10.1016/j.bbmt.2013.05.011 23707853

[40] Al-Homsi AS Feng Y Duffner U Al-Malki MM Goodyke A Cole K Borteaomib for the prevention and treatment of graft-versus-host disease after allogeneic hematopoietic stem cell transplantation. *Exp Hematol.* (2016) 44:771–7. 10.1016/j.exphem.2016.05.005 27224851

[41] Koreth J Kim HT Lange PB Poryanda SJ Reynolds CG Rai SC Bortezomib-based immunosuppression after reduced-intensity conditioning hematopoietic stem cell transplantation; randomized phase II results. *Haematologica.* (2018) 103:522–30. 10.3324/haematol.2017.176859 29326124 PMC5830392

[42] Al-Homsi AS Goodyke A McLAne M Abdel-Mageed S Cole K Cole K Post-transplation cyclopopsphamide and ixazomib combination rescues mice subjected to experimental graft-versu-host disease and is superior to either agent alone. *Biol Blood Marr Transplant.* (2017) 23:255–61. 10.1016/j.bbmt.2016.11.015 27888016

[43] Al-Homsi AS Cole K Muilenburg M Goodyke A Abidi M Duffner U Calcineurin and mTOR inhibitor-freep post-transplantation cyclophosphamide and bortezomib combination for graft-versus-host disease prevention after peripheral blood allogeneic hematopoietic stem cell transplantation: a phase I/II study. *Biol Blood Marr Transplant.* (2017) 23:1651–7. 10.1016/j.bbmt.2017.05.024 28549771

[44] Watkins BK Qayed M Bratrude B Betz K Sinclair S Suessmuth Y T cell costimulation blockade with CTLA4-Ig (abatecept) for acute Gvhd prevention in HLA matched and mismatched unrelated donor transplantation: results of the first phase 2 trial. *Biol Blood Marr Transplant.* (2019) 25(Suppl. 3):S51–52. 10.1016/j.bbmt.2018.12.129

[45] Park SC Jeen YT. Anti-integrin therapy for inflammatory bowel disease. *Worl J Gastroenterol.* (2018) 24:1868–80. 10.3748/wjg.v24.i17.1868 PMC5937204 29740202

[46] FlØisand Y Lundin KEA Lazarevic V Kristiansen JD Osnes LTN TjØnnfjord GE Targeting integrin α4β7 in steroid-refractory intesntinal graft-versus-host disease. *Biol Blood Marrow Transplant.* (2017) 23:172–5. 10.1016/j.bbmt.2016.10.009 27777142

[47] Pidala J Beato F Kim J Betts B Jim H Sagatys E *In vivo* IL-12/IL-23p40 neutralization blocks Th1/Th17 response after allogeneic hematopoietic cell transplantation. *Heamtologica.* (2018) 103:531–9. 10.3324/haematol.2017.171199 29242294 PMC5830373

